# Improving systems of care during and after a pregnancy complicated by hyperglycaemia: A protocol for a complex health systems intervention

**DOI:** 10.1186/s12913-020-05680-x

**Published:** 2020-09-01

**Authors:** D. MacKay, R. Kirkham, N. Freeman, K. Murtha, P. Van Dokkum, J. Boyle, S. Campbell, F. Barzi, C. Connors, K. O’Dea, J. Oats, P. Zimmet, M. Wenitong, A. Sinha, A. J. Hanley, E. Moore, D. Peiris, A. McLean, B. Davis, C. Whitbread, H. D. McIntyre, J. Mein, R. McDermott, S. Corpus, K. Canuto, J. E. Shaw, A. Brown, L. Maple-Brown, on behalf of the Diabetes Across the Lifecourse: Northern Australia Partnership, Vanya Webster, Sian Graham, Dianne Bell, Katarina Keeler, Chenoa Wapau, Martil Zachariah, Jennifer Barrett, Tara Dias, Kristina Vine, Bronwyn Davis, S. Chitturi, S. Eades, C. Inglis, K. Dempsey, M. Lynch, T. Skinner, R. Wright

**Affiliations:** 1 Menzies School of Health Research, Charles Darwin University, Darwin, Australia; 2 Royal Darwin Hospital, Darwin, Australia; 3 Baker Heart & Diabetes Institute Central Australia, Alice Springs, Australia; 4 Monash Centre for Health Research and Implementation, School of Public Health and Preventive Medicine, Monash University, Melbourne, Australia; 5 College of Nursing and Midwifery, Charles Darwin University, Cairns, Australia; 6 Top End Health Service, Northern Territory Department of Health, Darwin, Australia; 7 Population School of Health Research, University of South Australia, Adelaide, Australia; 8 Melbourne School of Population and Global Health, University of Melbourne, Melbourne, Australia; 9 Department of Diabetes, Central Clinical School, Monash University, Melbourne, Australia; 10 Apunipima Cape York Health Council, Bungalow, Australia; 11 Cairns and Hinterland Hospital and Health Service, Cairns, Australia; 12 Department of Nutritional Sciences, Faculty of Medicine and the Dalla Lana School of Public Health, The University of Toronto, Toronto, Canada; 13 Aboriginal Medical Services Alliance Northern Territory, Darwin, Australia; 14 The George Institute for Global Health, Sydney, Australia; 15 Mater Medical Research Institute, University of Queensland, Brisbane, Australia; 16 Wuchopperen Health Service, Cairns, Australia; 17 Centre for Chronic Disease Prevention, Australian Institute of Tropical Health and Medicine, James Cook University, Cairns, Australia; 18 Danila Dilba Health Service, Darwin, Australia; 19 South Australian Health and Medical Research Institute, Adelaide, Australia; 20 Aboriginal Health Domain, Baker IDI Heart and Diabetes Institute, Melbourne, Australia; 21 Faculty of Health and Medical Science, University of Adelaide, Adelaide, Australia

**Keywords:** diabetes in pregnancy, gestational diabetes, type 2 diabetes in pregnancy, health systems, healthcare delivery, health services, mixed methods evaluation, Indigenous Australian, Aboriginal, Torres Strait Islander

## Abstract

**Background:**

Many women with hyperglycaemia in pregnancy do not receive care during and after pregnancy according to standards recommended in international guidelines. The burden of hyperglycaemia in pregnancy falls disproportionately upon Indigenous peoples worldwide, including Aboriginal and Torres Strait Islander women in Australia. The remote and regional Australian context poses additional barriers to delivering healthcare, including high staff turnover and a socially disadvantaged population with a high prevalence of diabetes.

**Methods:**

A complex health systems intervention to improve care for women during and after a pregnancy complicated by hyperglycaemia will be implemented in remote and regional Australia (the Northern Territory and Far North Queensland). The Theoretical Domains Framework was used during formative work with stakeholders to identify intervention components: (1) increasing workforce capacity, skills and knowledge and improving health literacy of health professionals and women; (2) improving access to healthcare through culturally and clinically appropriate pathways; (3) improving information management and communication; (4) enhancing policies and guidelines; (5) embedding use of a clinical register as a quality improvement tool. The intervention will be evaluated utilising the RE-AIM framework at two timepoints: firstly, a qualitative interim evaluation involving interviews with stakeholders (health professionals, champions and project implementers); and subsequently a mixed-methods final evaluation of outcomes and processes: interviews with stakeholders; survey of health professionals; an audit of electronic health records and clinical register; and a review of operational documents. Outcome measures include changes between pre- and post-intervention in: proportion of high risk women receiving recommended glucose screening in early pregnancy; diabetes-related birth outcomes; proportion of women receiving recommended postpartum care including glucose testing; health practitioner confidence in providing care, knowledge and use of relevant guidelines and referral pathways, and perception of care coordination and communication systems; changes to health systems including referral pathways and clinical guidelines.

**Discussion:**

This study will provide insights into the impact of health systems changes in improving care for women with hyperglycaemia during and after pregnancy in a challenging setting. It will also provide detailed information on process measures in the implementation of such health system changes.

## Background

Hyperglycaemia in pregnancy, encompassing gestational diabetes mellitus (GDM), pre-existing type 2 diabetes (T2DM) and overt (likely type 2) diabetes in pregnancy, is associated with adverse health outcomes for mother and child, both in the peripartum period and long-term [1, 2]. The International Federation of Gynecology and Obstetrics (FIGO) recently identified improving systems of care for women with hyperglycaemia in pregnancy, particularly during the postpartum period, as a research priority [3]. The period during and after pregnancy is an opportune time to optimise maternal health, which is in turn an important strategy to reduce the risk of adverse outcomes in any future pregnancy. International guidelines provide recommendations for the care of women with hyperglycaemia in pregnancy, including postpartum glucose screening and counselling regarding contraception [4, 5]. However, there are significant gaps in implementing care which meets these recommendations, with a staggering 75-80% of women lost to follow-up [3], and an average of only 33% of women internationally completing postpartum glucose testing following GDM [6].

An estimated 16.9%, or 21.4 million, live births around the world are complicated by hyperglycaemia each year [7]. This burden falls disproportionately upon Indigenous women globally [8, 9]. In Australia, Aboriginal and Torres Strait Islander women are 10 times more likely to have pre-existing T2DM in pregnancy, and 1.5 times more likely to develop GDM [10, 11]. GDM is a strong predictor of future T2DM [12]; the risk of progressing to T2DM following GDM is fourfold greater for Aboriginal and Torres Strait Islander women than non-Indigenous women in Australia [13]. Children exposed to hyperglycaemia *in utero* are at risk of developing T2DM at an early age, which is an issue of increasing concern for the health of Aboriginal and Torres Strait Islander Australians [14, 15]. The consequences of hyperglycaemia in pregnancy contribute substantially to the epidemic of diabetes, and thus to the 10-year gap in life expectancy between Aboriginal and Torres Strait Islander peoples and non-Indigenous Australians [16, 17]. To address disparities in health outcomes between Aboriginal and Torres Strait Islander and non-Indigenous Australians, there is an urgent need to reduce risk as early as possible in the life course.

Prior interventions to improve care for women with hyperglycaemia in pregnancy have predominantly focussed on increasing the proportion of women attending glucose screening after GDM. Single component interventions, such as patient education programs, postpartum reminder systems or use of checklists, have not consistently demonstrated improvements [18–21]. This contrasts with multi-component interventions, which have achieved postpartum glucose screening rates as high as 95.8% [22–25]. Measures utilised in multi-component interventions include instituting protocol-based care, reminder systems, introduction of a nurse navigator, and education for healthcare providers and women. The impact of such intervention components is yet to be demonstrated in the regional and remote Australian context, where there are multiple barriers to implementing guideline-based recommendations for the care of women with hyperglycaemia in pregnancy. These barriers include a disproportionate burden of socioeconomic disadvantage, population mobility, geographic remoteness, high turnover of clinical staff, and fragmentation between service providers [26].

The Northern Territory Diabetes in Pregnancy (DIP) Partnership formed in 2011 between health service providers, policymakers and researchers to improve the care of women with hyperglycaemia in pregnancy. The Partnership expanded in 2016 to include the region of Far North Queensland, and more recently to consider the intergenerational impact of diabetes, and thus is now the “Diabetes Across the Lifecourse: Northern Australia Partnership” (“the Partnership”). Previous work of the Partnership has included improvements in antenatal service delivery for women with hyperglycaemia in pregnancy in the Northern Territory [26] and the establishment of the DIP Clinical Register [27].

Building on this previous work we have developed a multi-component health systems intervention to improve care for women across regions of northern Australia during and after a pregnancy complicated by hyperglycaemia. Components, which include reminder systems and health practitioner education, have been selected based on evidence for improving care of women with hyperglycaemia in pregnancy in other contexts [23, 25]. An additional component of our intervention is use of the DIP Clinical Register as a recall and continuous quality improvement tool. Clinical registers have been widely utilised, including in regional and remote Australia, to improve systems of care, clinical follow-up and health outcomes for chronic conditions [28, 29]. The Partnership has undertaken formative research with health professionals and stakeholders to identify gaps in care, which contributed to further refining our intervention design [26, 30, 31].

This protocol describes the planned implementation of our health systems intervention, in accordance with the Template for Intervention Description and Replication (TIDieR) [32] and the Revised Standards for QUality Improvement Reporting Excellence (SQUIRE 2.0) (Supplementary Materials) [33]. These frameworks both provide guidance intending to improve the completeness of reporting, and thus replicability, of healthcare interventions, with SQUIRE 2.0 specifically focussing on health service improvement.

## Methods

### Aim

To develop, implement and evaluate a health systems intervention to improve models of antenatal and postpartum care for women with hyperglycaemia in pregnancy in regional and remote Australia.

### Design

This study will use a cross-sectional before-and-after design to assess the impacts of the health systems intervention.

#### Theoretical Framework

Formative work was conducted with health professionals using the Theoretical Domains Framework (TDF) [34], identifying barriers to the implementation of care according to local guidelines [35, 36] for women during and after a pregnancy complicated by diabetes in remote and regional Australia. The TDF describes 12 domains covering the main factors that influence health practitioner clinical behaviour and behaviour changes: knowledge; skills; social/professional role and identity; beliefs about capabilities; beliefs about consequences; motivation and goals; memory, attention and decision processes; environmental context and resources; social influences; emotion; behavioural regulation; and nature of the behaviours. Use of these domains enables identification of a wide range of potential barriers to implementation of improvements in health systems, facilitating development of multiple potential intervention components to overcome the barriers identified.

Detailed barriers and enablers identified by this formative work have been previously reported [31, 37]. In brief, multiple factors impacting on health service delivery were identified, including fragmentation of care, gaps in communication and a lack of clarity regarding healthcare provider responsibilities for components of care such as screening and post-partum follow-up [37]. Further details regarding major barriers are described in Table 1. Opportunities to improve care were also identified, including enhancing education and support for health professionals and improving communication pathways [37].

**Table 1:** Barriers to implementing healthcare according to local guidelines for women during and after a pregnancy complicated by diabetes in regional and remote Australia, identified in formative work with healthcare professionals and stakeholders

System factors	Lack of clarity around roles of healthcare providers in administering diabetes screening tests and providing follow-up care in the postpartum period Insufficient involvement of medical specialists Disjointed communication pathways between hospital and primary care Inconsistent access to electronic health records Reliance on handheld medical record, which women may not bring to appointments Siloed approaches to provision of care High staff turnover Small Aboriginal health workforce Lack of systematic processes for referrals Unavailability of transport for women to attend for postpartum care Reported low numbers of women presenting for preconception counselling Requirement for many women to travel large distances to access care, with reluctance to leave other children behind Long waiting times at hospital clinics
Healthcare practitioner factors	Inconsistency in use of local guidelines Low health practitioner confidence in delivering care Lack of consistency of practitioner knowledge regarding criteria for screening for hyperglycaemia in early pregnancy
Patient factors	Prioritising needs of family over women’s own postpartum health Low perceived future risk of T2DM amongst women Limited time or motivation for women to attend for postpartum care

#### Setting

The Northern Territory (NT) and Far North Queensland (FNQ) encompass a geographic area of over 1.6 million square kilometres, including numerous remote islands. The region is sparsely populated, with approximately 500,000 inhabitants [38–40]. Approximately 22.5% of the population identifies as Aboriginal and/or Torres Strait Islander peoples, compared with 3.2% across Australia [38–41]. There is a high degree of cultural diversity, with over 200 languages spoken [40]. There are approximately 7000 births across the region annually [11, 38, 39]. In the NT in 2015, 11.9% of births to all women were complicated by GDM and 1.7% by pre-existing diabetes; for Aboriginal women, these rates were 15.4% and 4.1% respectively [11]. While the official numbers of births affected by diabetes in FNQ are not publicly reported, an audit of births to Aboriginal and Torres Strait Islander women showed the prevalence of GDM and T2DM to be 14.2% and 2.3% respectively [42].

**Fig. 1:**
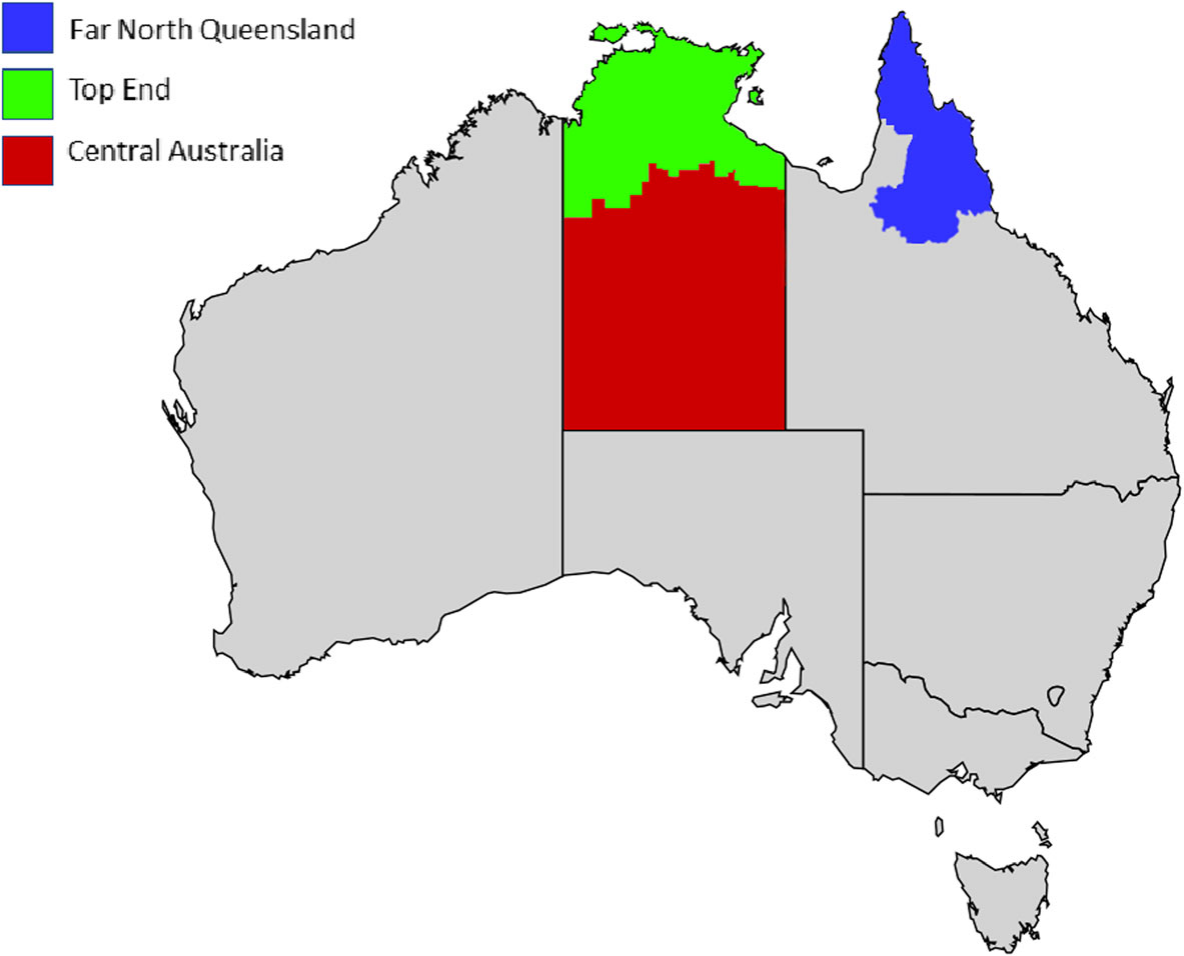
Study regions for a health systems intervention to improve care for women during and after a pregnancy complicated by diabetes. Adapted from: ‘Australia map, States.svg’ by Lokal_Profil available at https://commons.wikimedia.org/wiki/File:Australia_map,_States.svg under CC BY-SA 2.5. Full terms at https://creativecommons.org/licenses/by-sa/2.5/deed.en; ‘Australian Northern Territory location map.svg’ by NordNordWest available at https://commons.wikimedia.org/wiki/File:Australia_Northern_Territory_location_map.svg under CC-BY-SA-3.0-DE. Full terms at https://creativecommons.org/licenses/by-sa/3.0/de/deed.en; ‘Qld region map 2.png’ available at https://commons.wikimedia.org/wiki/File:Qld_region_map_2.PNG under CC BY-SA 3.0. Full terms at https://creativecommons.org/licenses/by-sa/3.0/deed.en

This health systems intervention will be conducted across tertiary, secondary and primary healthcare centres, including government and Aboriginal community-controlled organisations, throughout three study regions (Central Australia and Top End, within the NT; and FNQ) (Figure 1).

#### Participants

Health professionals across the NT and FNQ who are involved in the care or care coordination of women with hyperglycaemia in pregnancy.

#### Procedures

Formative focus groups conducted with health professionals in 2016-17 led to the identification of five key models of care components:

1. Increasing workforce capacity, skills and knowledge and improving the health literacy of health professionals and women.
2. Improving access to healthcare through culturally and clinically appropriate pathways.
3. Improving information management and communication.
4. Enhancing policies and guidelines.
5. Embedding the NT and FNQ Diabetes in Pregnancy Clinical Register [27] within the models of care as a continuous quality improvement tool.

#### Implementation Activities

**Fig. 2:**
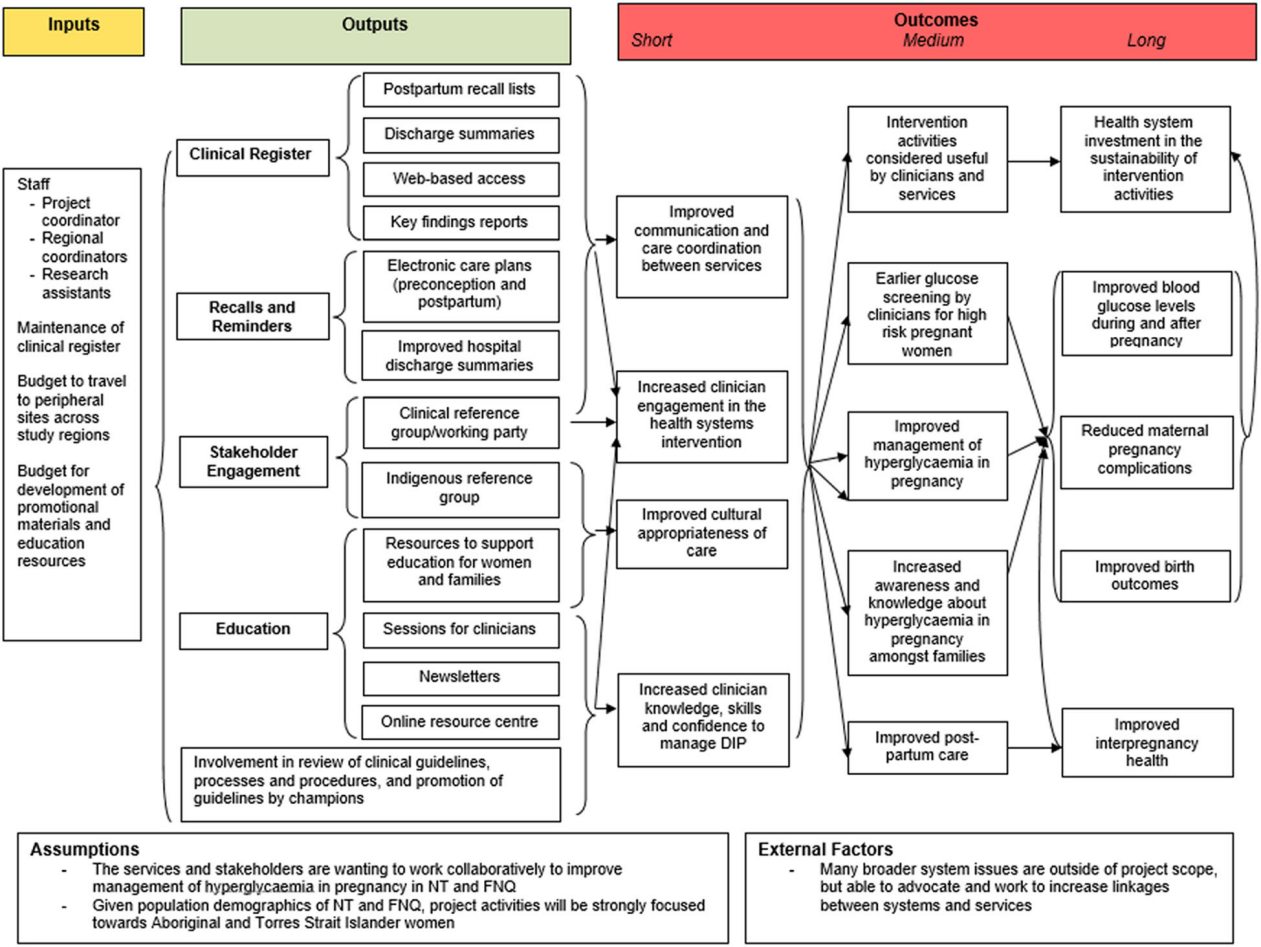
Logic model for a health systems intervention to improve care for women during and after a pregnancy complicated by diabetes

The above components have guided the development of implementation activities with local healthcare professionals and stakeholders to address identified barriers (Table 2; Figure 2). A project coordinator will lead implementation of these activities in each study region, with support from the central project office. Activities will be implemented across primary, secondary and tertiary level services throughout each study region, aiming to reach all health services across the regions providing care to women with hyperglycaemia in pregnancy. Due to differences in health systems between the study regions, it is anticipated that there will be context-specific variations in implementation activities across regions. Delivery of activities will be recorded by implementation staff in an Activity Log, including nature of activity, study region, Models of Care component addressed, number of attendees (where relevant), involvement of other organisations and feedback.

**Table 2:** Implementation activities to improve care for women during and after a pregnancy complicated by hyperglycaemia

Activity	Models of Care Components	TDF Domain	Procedure	Materials	Delivered by	Mode of delivery	Region (TE, CA, FNQ, All)
**Education for healthcare providers**	1, 2, 3, 4	Knowledge; professional role; beliefs about consequences; beliefs about capabilities	Develop an education calendar across each region to plan and deliver educational activities to healthcare practitioners, aligning with educational activities of other regional healthcare organisations (e.g. primary care networks) where possible, with invitation of healthcare providers through healthcare networks Selected sessions will be recorded and delivered online to enable access for practitioners unable to attend, with information for access distributed and promoted through Partnership networks and health practitioner organisations Newsletters to be distributed to healthcare providers and stakeholders through Partnership networks Annual symposium, with healthcare providers and other stakeholders invited through Partnership networks	Presentations, Newsletters, Workshops, Online resources (videos, podcasts)	Educational materials, including presentations and text-based materials, will be developed by project staff, with input from clinical experts (endocrinologists, diabetes nurse practitioner and educators, primary care practitioners) and Indigenous reference group Education sessions delivered by clinicians and project staff	Face-to-face Online Email Teleconference	All
**Postpartum care plans and reminders**	2, 3, 5	Memory, attention, decision-making	Develop postpartum care plans and reminders to align with and bridge to existing Chronic Disease Care Plan after pregnancy and embed within primary healthcare electronic health record, to prompt healthcare provider recall of women at recommended timepoints for review based on guidelines	Electronic primary care health record	Implementation team in collaboration with health services staff, with input from clinical reference group	Electronic primary care health record	TE, CA
**Preconception care plans**	2, 3, 5	Memory, attention, decision-making	Develop pre-conception care plans and embed within primary healthcare electronic health record, to prompt healthcare provider to ensure recommended pre-conception care is delivered to women with pre-existing diabetes based on guidelines	Electronic primary care health record	PhD student with input from clinical reference group	Electronic primary care health record	CA
**Indigenous reference group**	2, 3	Social influences	Form an Indigenous reference group to provide input regarding priority-setting, resource development and implementation, by inviting Indigenous women with an interest in hyperglycaemia in pregnancy, meeting three times per year and feeding back to investigators and project staff	Advice from Director of Aboriginal Programs, Menzies School of Health Research	Coordinated by Indigenous implementation team member	Face-to-face, with email communication between meetings	TE, CA
**Clinical reference group**	2, 3	Social influences; motivation and goals	Ongoing facilitation of a reference group of clinicians to provide input regarding priority-setting, resource development and implementation, by inviting interested clinicians through Partnership networks, to meet annually and feed back to investigators and project staff	Presentations; meetings; circulation of documents/ resources for comment	Coordinated by implementation team	Face-to-face, with clinicians provided with email address to provide feedback between meetings	TE, CA
**Working group**	2, 3	Social influences; motivation and goals	Form a working group with representatives from partner organisations to provide input regarding priority-setting, resource development and implementation, as well as opportunity for promotion of educational opportunities	Presentations; meetings; circulation of documents/ resources for comment	Coordinated by implementation team	Face-to-face meetings alternate months	FNQ
**Resource development**	1, 2, 3	Environmental context and resources	Development of culturally appropriate resources to assist healthcare providers in discussions with women about health after a pregnancy complicated by diabetes	Postpartum discharge brochure	Discharge brochure developed by implementation team with input from clinical experts	Paper-based	All
**Aggregate DIP Clinical Register reports**	1, 3, 5	Knowledge; motivation and goals	Produce de-identified aggregate postpartum reports from the DIP Clinical Register six-monthly distribute to healthcare providers and stakeholders to enable quality improvement activities	DIP Clinical Register	Implementation team	Email	All
**Local DIP Clinical Register reports**	1, 3, 5	Memory, attention, decision-making	Produce local postpartum reports with identifiable data from the DIP Clinical Register six-monthly and distribute to healthcare providers to aid in quality improvement activities and recall of women	DIP Clinical Register	Implementation team	Email	TE, CA
**Modified discharge summaries**	3, 5	Memory, attention, decision-making	Review and amend current discharge summary templates for discharge from hospital after delivery, to include options and prompts to facilitate communication of follow-up plans between hospital and primary care providers	Discharge summaries	Implementation team	Within electronic discharge summary	TE, CA
**Postpartum summary**	3, 5	Memory, attention, decision-making	Generate postpartum diabetes in pregnancy summary using DIP Clinical Register data and distribute to healthcare providers, including reminder for postpartum screening	DIP Clinical Register	Implementation team	Letter	FNQ
**Postpartum screening reminder letters**	3, 5	Memory, attention, decision-making	Generate letters to healthcare providers using DIP Clinical Register data to prompt recall of women for recommended postpartum glucose check if check not recorded within six months postpartum	DIP Clinical Register	Implementation team	Letter	FNQ
**Promotion of postpartum guidelines by champions**	1, 4	Social influences	Champions identified through engagement with Partnership activities, and upskilled regarding use of local guidelines through Partnership educational activities and publications	Local clinical guidelines – CARPA (CA, TE), QCG (FNQ)	Implementation team	Face-to-face, email	All

Models of Care Components: 1 – Increasing workforce capacity, skills and knowledge and improvement in the health literacy of health professionals and women; 2 – Improving access to culturally and clinically appropriate healthcare; 3 – Improving information management and communication; 4 – Enhancing policy and guidelines; 5 – Embedding the Diabetes in Pregnancy Clinical Register as a component with the Models of Care

*Abbreviations*: *CA* Central Australia, *CARPA* Central Australian Rural Practitioners Association (2017), *DIP* Diabetes in Pregnancy, *FNQ* Far North Queensland, *QCG* Queensland Clinical Guidelines (2015), *TDF* Theoretical Domain Framework, *TE* Top End, the Partnership – Diabetes Across the Lifecourse: Northern Australia Partnership

Activities and associated materials will be modified throughout the delivery of the project based on feedback from health professionals, project implementers and other stakeholders. The interim evaluation (see below) will provide the main opportunity for feedback and modifications; additional opportunities will be facilitated through regular meetings with stakeholder groups, including a Clinical Reference Group and Indigenous Reference Group in the NT, and a Partnership Working Group in FNQ.

Key implementation activities can be grouped into: educational sessions and resources; recall and reminder systems to assist with follow-up; establishing (in FNQ) and expanding (in NT) the use of the Diabetes in Pregnancy Clinical Register; and stakeholder engagement and consultation (Figure 2). There is substantial overlap between these activities, and many activities align with several of the Models of Care components. Further detail is provided in Table 2.

##### Educational Sessions and Resources

Implementation staff will collaborate with expert clinicians in the development and delivery of education to health professionals involved in the antenatal and postpartum care of women with hyperglycaemia in pregnancy. Face-to-face and online educational sessions will be designed to reach relevant healthcare providers, with sessions promoted through professional networks and health services. Education will promote evidence-based care according to local guidelines. Educational materials will be made available to interested health professionals for distribution to networks. Key messages will be highlighted in regular newsletters distributed to health professionals and stakeholders (see below, Stakeholder Engagement and Consultation).

Additional educational materials which are culturally appropriate for Aboriginal and Torres Strait Islander women with a pregnancy complicated by diabetes will be developed in collaboration with the Partnership’s Indigenous Reference Group and provided to healthcare services for distribution to women.

##### Recall and Reminder Systems

Implementation staff will collaborate with health services to improve systems and enhance recall of women with a pregnancy complicated by diabetes. This will include developing and embedding appropriate postpartum and preconception care plans with reminder systems in primary care electronic health records; improvement of discharge summary templates following hospital discharge; providing postpartum summaries with guideline-based recommendations for ongoing care; and generating postpartum lists from the Diabetes in Pregnancy Clinical Register of women with a recent pregnancy complicated by hyperglycaemia for distribution to primary care services (see below, Diabetes in Pregnancy Clinical Register).

##### Diabetes in Pregnancy Clinical Register

Implementation of the Diabetes in Pregnancy (DIP) Clinical Register in the NT has previously been described in detail [27, 43]. In brief, the register was established in the NT in 2011 and documented all consenting women residing in the NT aged 16 years and over with any type of hyperglycaemia in pregnancy, referred by a health professional.

In the current health systems intervention, the implementation team will collaborate with health services to establish the DIP Clinical Register in FNQ, and expand its use in the NT. This will include streamlining referral processes by embedding referrals within electronic health records of health services where possible. Functions of the DIP Clinical Register include producing and distributing local postpartum reports with details of women attending specific primary healthcare services who have recently given birth, to aid with recall of women and quality improvement activities. In addition, de-identified aggregate reports including all women across the NT and FNQ are produced and distributed to healthcare providers and stakeholders as a quality improvement and epidemiological tool. Implementation staff will work with healthcare staff to use findings of aggregated DIP Clinical Register reports as quality improvement tools to inform changes in models of care and improve integration between primary and hospital-based care.

##### Stakeholder Engagement and Consultation

Ongoing collaboration with stakeholders will be essential for implementation of this health systems intervention. The Partnership has established reference groups (a Clinical Reference Group and an Indigenous Reference Group in the NT, and a Partnership Working Group in FNQ), who will meet regularly throughout the health systems intervention to provide feedback on implementation activities and guide ongoing priorities of the intervention. Additional consultation will occur frequently with key health service representatives and champions. The Partnership will produce regular newsletters to provide educational messages and update health practitioners and stakeholders on project activities.

#### Evaluation

This health systems intervention will be evaluated using the five dimensions of the RE-AIM framework: reach (the proportion and representativeness of participants relative to target population), effectiveness (impacts of the program), adoption (uptake of the intervention), implementation (the extent to which the intervention was delivered as intended) and maintenance (the extent to which the intervention is institutionalised into routine organisational practices and policies) [44–46].

Evaluation indicators for each phase of the evaluation have been developed and structured within the RE-AIM framework (Tables 3 and 4) [44]. Outcome measures include changes between baseline and post-intervention in indicators of care provided to women, including: proportion of high risk women receiving recommended glucose screening in early pregnancy; first and third trimester HbA1c in women with T2DM; and proportion of women receiving recommended postpartum care (glucose testing, breastfeeding, weight management, smoking education and discussion or prescription of contraception). We will also assess changes in health practitioner confidence in providing care, knowledge and use of relevant guidelines and referral pathways, and perception of care coordination and communication systems; and changes to health systems including referral pathways and clinical guidelines.

**Table 3:** Indicators for the interim evaluation of a health systems intervention to improve care during and after a pregnancy complicated by diabetes

RE-AIM	Indicator	Data Sources
**R**each - *levels of participation and characteristics of participants*	•Awareness of the Partnership and associated activities •Role of participant and level of engagement with hyperglycaemia in pregnancy clients	Health professionals Implementation team, enablers, champions
**E**ffectiveness - *positive and negative consequences of the intervention*	•Perceived level of effectiveness of resources/activities for improving management of hyperglycaemia in pregnancy •Acknowledgement of factors that contribute to effectiveness/explanation of varying levels of effectiveness	Health professionals Implementation team, enablers, champions
**A**doption - *proportion and representativeness of settings and providers who have adopted the intervention (or components of it)*	•Knowledge and/or resources have been adopted in practice or intended to be adopted (i.e. improved management practices adopted such as – follow-up plans, OGTT’s, Chronic Disease Management Plans) •Issues related to not taking up Partnership activity opportunities and/or not implementing related activities	Health professionals Implementation team, enablers, champions
**I**mplementation - *the intervention is delivered as intended*	•Extent that the Models of Care components*/implementation activities are being delivered as planned/expected, by whom and when •Adaptations made to original implementation plan	Implementation team, enablers, champions
**M**aintenance - *practice or policy becomes routine and part of everyday culture and norms*	•Extent that the Models of Care components*/implementation activities have been embedded into regular practice •Intention to continue new practices beyond the project’s funding cycle	Health professionals Implementation team, enablers, champions

“The Partnership” - the Diabetes Across the Lifecourse: Northern Australia Partnership; OGTT – 75 gram oral glucose tolerance test

*Models of Care Components: 1 – Increasing workforce capacity, skills and knowledge and improvement in the health literacy of health professionals and women; 2 – Improving access to culturally and clinically appropriate healthcare; 3 – Improving information management and communication; 4 – Enhancing policy and guidelines; 5 – Embedding the Diabetes in Pregnancy Clinical Register as a component with the Models of Care

**Table 4:** Indicators for the final evaluation of a health systems intervention to improve care during and after a pregnancy complicated by diabetes

Objective	Final evaluation question addressed*	Indicator	Data source	Data collection
*REACH*				
Increase (FNQ) and sustain (NT) engagement of clinicians with the project	3	Health practitioner awareness of Partnership and activities	Health professionals	Interviews
	3	Health practitioner attendance at Partnership education events	Project activity log	Surveys
	3	Use of project online health professional educational resources	Website	Activity log Metrics from website
Improve health practitioner awareness of DIP Clinical Register	3	Health practitioner awareness of DIP Clinical Register	Health professionals DIP Clinical Register	Interviews Surveys
Increase (FNQ) and sustain (NT) coverage of the DIP Clinical Register	1	DIP Clinical Register coverage; trajectory of coverage over time	Comparison of DIP Clinical Register with health service data	Health service reports
Determine the number and characteristics of women accessing and not accessing antenatal care		Number of women accessing antenatal care, including number and timing of visits	Health service electronic health records DIP Clinical Register Health service reports^1^	Audit
*EFFECTIVENESS*				
Enhance support for health practitioners	1	Health practitioner perception of support	Health professionals	Interviews
	4	Health practitioner and champion reports of which activities have been useful in enhancing support	Champions	Surveys
Increase health practitioner awareness of and confidence in managing hyperglycaemia in pregnancy	1	Health practitioners perceived knowledge and confidence, and changes from Partnership formative work	Health professionals Health service electronic health records	Interviews Surveys
	1	Rates of completion of recommended glucose screening in early pregnancy for high risk women	DIP Clinical Register Formative DIP Models of Care work	Audit
Earlier hyperglycaemia in pregnancy screening women at high risk	1	Rates of completion of recommended early pregnancy screening for high risk women	DIP Clinical Register	Audit
Improved blood glucose levels for women with diabetes in pregnancy	2	Mean first- and third-trimester HbA1c and changes over time	DIP Clinical Register	Audit
Improved birth and neonatal outcomes	2	Gestational age at delivery Mode of delivery Birth weight Large for gestational age Small for gestational age Macrosomia Neonatal obstetric trauma Neonatal hypoglycaemia requiring treatment Neonatal special care admission 5-minute APGAR score less than 5 Neonatal jaundice requiring treatment Neonatal respiratory distress	DIP Clinical Register	Audit
Improve health practitioners’ awareness of postpartum guidelines	1	Health practitioner awareness of guidelines and changes over time	Health professionals Champions	Interviews Surveys
Improve postpartum management, according to guidelines, following diabetes in pregnancy	2	Proportion of women completing postpartum glucose testing Postpartum weight, body mass index, waist circumference Proportion of women breastfeeding Proportion of women smoking Proportion of women prescribed contraception, or who have discussed contraception with a health practitioner Changes over time in all indicators	Electronic health records DIP Clinical Register	Audit
Enhance communication between primary healthcare and hospital services	1	Health practitioner perception of communication between primary healthcare and hospital services	Health professionals	Interviews
	3	Health practitioner and champion reports of which activities have contributed to changes	Champions	Surveys
Improve referral pathways and care coordination for services caring for women with hyperglycaemia in pregnancy	1	Health practitioner knowledge of referral pathways	Health professionals	Interviews
	1	Health practitioner perception of improvements in care coordination	Champions	Surveys
	3	Health practitioner and champion reports of which activities have contributed to changes		
Improve discharge processes	1	Health practitioner perception of usefulness of discharge summaries	Health professionals	Interviews
	1	Health practitioner and champion perception of impact of discharge processes on postpartum care	Champions	Surveys
*ADOPTION*				
Enhance referrals to DIP Clinical Register	1	DIP Clinical Register coverage	Comparison of DIP Clinical Register with health service data Health professionals	Health service reports^1^ Interviews Surveys
	3	Health service perceptions of referral process		
Improve practitioner use of DIP Clinical Register	1	Health practitioner use of DIP Clinical Register and reports	Health professionals	Interviews
	3	Health practitioner reports of which aspects of reports are useful in practice	DIP Clinical Register	Surveys External use of DIP Clinical Register website, e.g. website metrics
Identify enablers and barriers impacting on adoption of project activities	3	Health practitioner, implementer and champion reports of enablers and barriers	Health professionals Implementers Champions	Interviews
Determine acceptability and value of project activities •Are project activities socially appropriate/ acceptable? •What is the social importance of project outcomes? •Which project activities are perceived as valuable?	4	Health practitioner, implementer and champion perceptions of acceptability and value of project activities	Health professionals Implementers Champions Women	Interviews
*IMPLEMENTATION*				
Determine if project activities have been delivered as intended	3	Proportion of planned activities delivered	Project activity log Implementers	Interviews Audit of activity log
Determine if project activities have been adapted, e.g. to fit local needs	3	Adaptations of planned activities and rationale	Implementers	Interviews
Identify enablers and barriers impacting on implementation of project activities	3	Enablers and barriers as identified by implementation team	Implementers Health professionals Champions	Interviews
*MAINTENANCE*				
Sustain DIP Clinical Register through integration with other structures	5	Health practitioners and services perceptions of sustainability of the DIP Clinical Register Resources required and cost of maintaining DIP Clinical Register	Health professionals Champions Implementers Activity log	Interviews Surveys Cost-consequences analysis
Identify project activities sustainable beyond project completion, and method for funding or integration into existing services	5	Health professional, champion and implementer perception of sustainability of project activities Resources required for project activity sustainability	Health professionals Champions Implementers Activity log	Interviews Cost-consequences analysis

*Evaluation questions: 1. To what degree has the health systems intervention improved systems of care during and after a pregnancy complicated by hyperglycaemia?; 2. To what degree has the health systems intervention improved maternal and neonatal outcomes during and after a pregnancy complicated by hyperglycaemia?; 3. If improvements are demonstrated, which activities of the health systems intervention have contributed to this improvement, and what enablers and barriers have impacted on the success of these activities?; 4. Are activities considered socially valid by healthcare providers, champions and stakeholders?; 5. How do the resources required for project activities balance against the benefits?; 6. How can the Partnership support the continuation of successful project activities after completion of the health systems intervention?

*Abbreviations*: *NT* Northern Territory, *FNQ* Far North Queensland

^1^*NT* Midwives’ Data Collection, *FNQ* Queensland Health Case Mix reports

Changes in birth and neonatal outcomes will be assessed to determine the impact of health system changes, including: gestational age at delivery; mode of delivery; birth weight; large for gestational age; small for gestational age; macrosomia; neonatal obstetric trauma; neonatal hypoglycaemia requiring treatment; neonatal special care admission; 5-minute APGAR score less than 5; neonatal jaundice requiring treatment; and neonatal respiratory distress.

The health systems intervention will be evaluated across multiple levels (individual health practitioner, clinic, health system, community), with data collection occurring at three timepoints:

1. Baseline, prior to implementation of the intervention, to enable comparison across indicators pre- and post-intervention.
2. A qualitative interim evaluation, to be conducted at least 12 months after delivery of intervention activities has commenced across all regions, focusing on identifying and exploring barriers and enablers of implementation of and engagement with the health systems intervention. Findings from this interim evaluation will inform modifications of the intervention, for the remainder of the implementation period.
3. A mixed-methods final evaluation of outcomes and processes of the health systems intervention, after completion of the implementation period. This evaluation will address whether the health systems intervention has improved systems of care for women during and after a pregnancy complicated by diabetes; which implementation activities have contributed to improvements; enablers and barriers impacting on the success of implementation activities; social validity of the health systems intervention (from the perspectives of healthcare providers, champions and stakeholders); and how implementation activities can be sustained beyond completion of the intervention. Quantitative data will include measures of care provided during and after pregnancy, as well as birth outcomes.

#### Data Collection

Data will be collected from four sources: interviews with healthcare providers and stakeholders; healthcare provider survey; cross-sectional pre- and post-intervention audits of electronic health records and the Diabetes in Pregnancy Clinical Register; and implementation operational documents. Six primary healthcare services, including one government and one community-controlled service in each of the three study regions, will be evaluation case study sites. Case study sites include both an urban and a remote service in each study region. Data will additionally be collected at the regional health service level, including regional referral hospitals and health service management.

##### Semi-Structured Interviews

Healthcare providers and stakeholders at each of the evaluation case study sites will be interviewed during both the interim and final evaluations. Additional interviews will be conducted with relevant stakeholders at the major referral hospital within each region (Top End: Royal Darwin Hospital; Central Australia: Alice Springs Hospital; FNQ: Cairns Hospital), and with policymakers and managers at the regional health service level, as well as with members of the implementation team. These interviews will be guided by a social constructionist epistemological perspective, utilising a phenomenological approach. Interview topics will include awareness of, engagement with and utility of activities of the health systems intervention, and the impact of intervention activities on practice. Interviews with implementation staff will additionally address process measures including barriers and facilitators to implementing intervention activities, any adaptations to implementation activities and the rationale and success for these adaptations. Interviews during the final evaluation will also explore healthcare provider confidence in providing care, perceptions of care coordination and communication and how these have changed during the health systems intervention; perceived impact of the intervention on care for women whose pregnancies are complicated by hyperglycaemia; and sustainability of implementation activities.

##### Survey

Healthcare providers in the study regions involved in the care or care coordination of women during and/or after a pregnancy complicated by hyperglycaemia will be invited through health professional networks to complete an online survey after completion of the implementation period. Participants will be asked about their usual practice in providing care for women during and after a pregnancy complicated by diabetes; confidence and support received in providing care; satisfaction with care pathways (e.g. access to specialist and allied health care) and communication between services; and awareness and engagement with activities of the health systems intervention. Response rates will be estimated by region and profession by comparing with number of professionals per region registered with relevant professional associations, where these numbers are available. Baseline survey data for comparison was previously collected during formative work [31, 37].

##### Audit of Electronic Health Records and Diabetes in Pregnancy Clinical Register

Primary care medical records and the DIP Clinical Register will be assessed using an independent sample, cross-sectional, pre- and post-intervention audit to determine the proportion of women receiving recommended care during and after pregnancy. In the NT, de-identified remote primary care medical records will be requested from NT Department of Health (DoH) for women with a birth due date in the final 12 months of implementation of the health systems intervention and who had a diagnosis of diabetes in pregnancy, and compared to baseline data already provided by NT DoH. In FNQ, data will be collected from the FNQ DIP Clinical Register, with baseline data including women giving birth in the first 12 months of the DIP Clinical Register’s inception and post-intervention data including women giving birth in the final 12 months of the intervention. Data from primary healthcare provider electronic health records at evaluation case study sites not covered by NT DoH records (Aboriginal Community-Controlled Health Services in NT, and all FNQ evaluation sites) will also be requested for women with hyperglycaemia in pregnancy giving birth within the above time periods. Independent variables collected will include: study region; age; ethnicity; and type of diabetes. Outcome variables will include: number and timing of clinic visits (antenatal and postpartum); completion of recommended glucose screening in early pregnancy for high risk women; mean first- and third-trimester HbA1c; first- and third-trimester smoking status; postpartum variables within 6 months of birth including completion of glucose screening (including 75 gram OGTT, fasting glucose, HbA1c, random glucose, any glucose measure), weight, body mass index, waist circumference, breastfeeding status, smoking status and contraception use (prescribed or discussed with health practitioner); and birth and neonatal outcomes (gestational age at delivery, mode of delivery, birth weight, large for gestational age, small for gestational age, macrosomia, neonatal obstetric trauma, neonatal hypoglycaemia requiring treatment, neonatal special care admission, 5-minute APGAR score less than 5, neonatal jaundice requiring treatment, neonatal respiratory distress).

##### Implementation Operational Documents

The intervention Activity Log will be reviewed, in addition to minutes of stakeholder meetings (including the Clinical and Indigenous Reference Groups) and attendance sheets and feedback forms from educational events.

#### Data Analysis

##### Qualitative Analyses

Qualitative analyses of interview transcripts and operational documents will employ a hybrid inductive-deductive method. The first round of coding will be an inductive analysis; the second round of coding will involve a deductive analysis utilising the pre-specified evaluation indicators. Themes will be clustered by study region, enabling comparison and contrast between regions. NVivo (version 12) will be used to assist the analysis processes.

##### Quantitative Analyses

Survey data will be compared with baseline data collected through a prior health professional survey across the study regions, conducted during formative work for this health systems intervention [31, 37]. Descriptive statistics will be reported. Changes between baseline and post-intervention for categorical variables will be analysed by Pearson’s Chi-squared test or Fisher’s exact test.

Data on outcome variables collected from the audit of electronic healthcare records and the DIP Clinical Register will be compared between baseline and post-intervention periods. Changes between pre- and post-intervention in categorical variables will be analysed using Pearson’s Chi-squared test; changes in continuous variables will be analysed using student’s *t*-test. Multivariable analysis will be performed using logistic regression for categorical and linear regression for continuous variables to assess relationships with independent variables, including age, ethnicity, and diabetes type.

All quantitative analyses will be performed using STATA 15.0 (StataCorp, College Station, Texas). The threshold for statistical significance will be defined as *p*<0.05 on two-tailed testing.

## Discussion

Our health systems intervention is the first to our knowledge which aims to improve care both during pregnancy and the postpartum period for women across the broad spectrum of hyperglycaemia in pregnancy, including GDM, pre-existing type 2 diabetes and overt diabetes in pregnancy. As described above, published work to date has focussed largely on improving postpartum glucose screening specifically in women with GDM [22–25]; despite widespread recommendations regarding provision of multidisciplinary care during and after pregnancy for women with pre-existing diabetes, a substantial knowledge gap regarding the evaluation of models of care for women with pre-existing diabetes has been identified by others [47]. Our health systems intervention is unique as it aims to improve care for women with all forms of hyperglycaemia in pregnancy, both during and after pregnancy. In addition, this intervention focuses on healthcare providers delivering care to a population with a high burden of disease in a complex setting.

The development of this health systems intervention from formative work conducted with health professionals is a strength of the project. This intervention aims to translate the knowledge of barriers and opportunities identified in our formative findings into tangible health service improvements. Previous work of the Partnership in the NT includes improving systems of care during pregnancy and implementing the NT DIP Clinical Register [26, 27]. These endeavours have resulted in the development of strong relationships between clinicians, policymakers and researchers. The current health systems intervention is strengthened through being informed by the input of these many stakeholders. Stakeholder collaboration will continue throughout implementation, further strengthening this intervention.

The mixed-methods evaluation, utilising multiple data sources, is another strength of this health systems intervention, enabling triangulation of findings. Quantitative audit data will demonstrate whether the intervention impacts on indicators of care provided to women during and after pregnancy, and on birth outcomes. Previous research has demonstrated the accuracy of routinely collected data regarding chronic conditions in electronic health records in remote Aboriginal communities, making this a useful resource for evaluation [48], together with use of our established DIP Clinical Register to assess pre- and post-intervention birth outcomes. The qualitative data will provide insights about acceptability and long-term feasibility of activities of the health systems intervention which will be essential information, both for planning sustainability of successful activities in the local setting and enabling replication in other settings.

The inclusion of multiple study regions, with different health systems operating across the regions and associated variation in organisational structure, policies and processes, is a challenge in this study, and will likely necessitate alterations in implementation activities depending on study region. However, this is also a strength of this intervention, providing an opportunity to assess how contextual factors of each region impact on the implementation and outcomes of the intervention. Information regarding contextual factors and their impact will form part of the findings of interest from this study.

The broad range of implementation activities that comprise this health systems intervention presents an additional challenge with regards to evaluating the impacts of these activities. This challenge is addressed through the use of multiple data sources within a structured evaluation framework, namely RE-AIM, which facilitates an in-depth exploration of the relative contributions of activities and their implementation to any observed outcomes.

Exploring the perspectives of Aboriginal and Torres Strait Islander women is essential in ensuring that healthcare is delivered in a way that meets these women’s needs and is culturally safe and appropriate. The Partnership’s Indigenous Reference Group is an important mechanism for ensuring voices of Aboriginal and Torres Strait Islander women are represented in this study. Additional planned work of the Partnership will ensure consumer perspectives are considered, namely those of Aboriginal and Torres Strait Islander women with a pregnancy complicated by hyperglycaemia, and inform future initiatives including greater engagement with consumers as participants of the intervention.

In conclusion, the Diabetes Across the Lifecourse: Northern Australia Partnership’s health systems intervention has the potential to improve care for women during and after a pregnancy complicated by hyperglycaemia and intervene as early as possible in the life course to improve the health of women and their children. This study is anticipated to lead to improvements in clinician knowledge and skills in the management of hyperglycaemia in pregnancy. More broadly, this study has significance for health systems policy and implementation, particularly in populations at high risk of hyperglycaemia in pregnancy and transmission of risk to the next generation, including Indigenous peoples worldwide.

## Supplementary information

**Additional file 1.** Revised Standards for Quality Improvement Reporting Excellence (SQUIRE 2.0) September 15, 2015

**Additional file 2.** The TIDieR (Template for Intervention Description and Replication) Checklist*

## Data Availability

Not applicable

## Abbreviations

BMI: Body mass index
CA: Central Australia
DIP: Diabetes in pregnancy
EHR: Electronic health record
FNQ: Far North Queensland
GDM: Gestational diabetes
HbA1c: Glycated haemoglobin A1c
NT: Northern Territory
OGTT: 75 gram oral glucose tolerance test
PANDORA: Pregnancy and Neonatal Diabetes Outcomes in Remote Australia study
T2DM: Type 2 diabetes
TE: Top End
