# Supplementary material for: Improving systems of care during and after a pregnancy complicated by hyperglycaemia: A protocol for a complex health systems intervention
Source: BMC Health Serv Res. 2020 Sep 1;20:814. doi: 10.1186/s12913-020-05680-x (PMC7461356; doi:10.1186/s12913-020-05680-x)
Supplement: Supplementary file 1 — Additional file 1. Revised Standards for Quality Improvement Reporting Excellence (SQUIRE 2.0) September 15, 2015 [file 12913_2020_5680_MOESM1_ESM.docx]

Revised Standards for Quality Improvement Reporting Excellence (SQUIRE 2.0) September 15, 2015

| Title and abstract | | |
| --- | --- | --- |
| 1. Title | Indicate that the manuscript concerns an initiative to improve healthcare (broadly defined to include the quality, safety, effectiveness, patient-centeredness, timeliness, cost, efficiency, and equity of healthcare) | Page 1 |
| 1. Abstract | a. Provide adequate information to aid in searching and indexing b. Summarize all key information from various sections of the text using the abstract format of the intended publication or a structured summary such as: background, local problem, methods, interventions, results, conclusions | Page 2 |
| Introduction | Why did you start? |  |
| 1. Problem and description | Nature and significance of the local problem | Pages 4-5 |
| 1. Available knowledge | Summary of what is currently known about the problem, including relevant previous studies | Page 5 |
| 1. Rationale | Informal or formal frameworks, models, concepts, and/or theories used to explain the problem, any reasons or assumptions that were used to develop the intervention(s), and reasons why the intervention(s) was expected to work | Pages 5-8 |
| 1. Specific aims | Purpose of this project and of this report | Page 6 |
| Methods | What did you do? |  |
| 1. Context | Contextual elements considered important at the outset of introducing the intervention(s) | Pages 7-8; Table 1 |
| 1. Intervention(s) | a. Description of the intervention(s) in sufficient detail that others could reproduce it b. Specifics of the team involved in the work | Pages 9-12; Table 2 |
| 1. Study of the intervention(s) | a. Approach chosen for assessing the impact of the intervention(s) b. Approach used to establish whether the observed outcomes were due to the intervention(s) | Pages 12-14 |
| 1. Measures | a. Measures chosen for studying processes and outcomes of the intervention(s), including rationale for choosing them, their operational definitions, and their validity and reliability b. Description of the approach to the ongoing assessment of contextual elements that contributed to the success, failure, efficiency, and cost c. Methods employed for assessing completeness and accuracy of data | Pages 15-17; Tables 3 and 4 |
| 1. Analysis | a. Qualitative and quantitative methods used to draw inferences from the data b. Methods for understanding variation within the data, including the effects of time as a variable | Pages 17-18 |
| 1. Ethical considerations | Ethical aspects of implementing and studying the intervention(s) and how they were addressed, including, but not limited to, formal ethics review and potential conflict(s) of interest | Page 22 |
| Results | What did you find? |  |
| 1. Results | a. Initial steps of the intervention(s) and their evolution over time (e.g., time-line diagram, flow chart, or table), including modifications made to the intervention during the project b. Details of the process measures and outcome c. Contextual elements that interacted with the intervention(s) d. Observed associations between outcomes, interventions, and relevant contextual elements e. Unintended consequences such as unexpected benefits, problems, failures, or costs associated with the intervention(s). f. Details about missing data | N/A |
| Discussion | What does it mean? |  |
| 1. Summary | a. Key findings, including relevance to the rationale and specific aims b. Particular strengths of the project | Pages 18-20 |
| 1. Interpretation | a. Nature of the association between the intervention(s) and the outcomes b. Comparison of results with findings from other publications c. Impact of the project on people and systems d. Reasons for any differences between observed and anticipated outcomes, including the influence of context e. Costs and strategic trade-offs, including opportunity costs | N/A |
| 1. Limitations | a. Limits to the generalizability of the work b. Factors that might have limited internal validity such as confounding, bias, or imprecision in the design, methods, measurement, or analysis c. Efforts made to minimize and adjust for limitations | Page 20 |
| 1. Conclusions | a. Usefulness of the work b. Sustainability c. Potential for spread to other contexts d. Implications for practice and for further study in the field e. Suggested next steps | Page 21 |
| Other information |  |  |
| 1. Funding | Sources of funding that supported this work. Role, if any, of the funding organization in the design, implementation, interpretation, and reporting | Page 23 |
